# Advances in Diagnosis and Treatment With Cholangiopancreatoscopy

**DOI:** 10.1111/den.70141

**Published:** 2026-03-30

**Authors:** Takeshi Ogura, Nga Nguyen Trong, Jayanta Samanta

**Affiliations:** ^1^ Pancreatobiliary Advanced Medical Center Osaka Medical and Pharmaceutical University Hospital Osaka Japan; ^2^ 2nd Department of Internal Medicine Osaka Medical and Pharmaceutical University Osaka Japan; ^3^ Endoscopy Center Osaka Medical and Pharmaceutical University Hospital Osaka Japan; ^4^ Gastroenterology Trong Nam Cancer Hospital Ha Noi Vietnam; ^5^ Department of Gastroenterology Post Graduate Institute of Medical Education and Research Chandigarh India

**Keywords:** biliary, ERCP, pancreas, peroral cholangioscopy, peroral pancreatoscopy

## Abstract

Endoscopic retrograde cholangiopancreatography (ERCP) remains the standard for diagnosing and treating pancreatobiliary diseases. Although non‐invasive imaging modalities have significantly improved, ERCP continues to play an indispensable role in clinical practice. However, ERCP has inherent limitations. Diagnostic challenges persist in cases of indeterminate biliary strictures, and stone extraction can be difficult when stones are large, impacted, or located within anatomically complex regions. To address these limitations, direct visualization of the bile and pancreatic ducts using peroral cholangioscopy (POCS) and peroral pancreatoscopy (POPS) has gained increasing attention. These techniques enable targeted biopsies under direct vision, thereby enhancing diagnostic accuracy for indeterminate strictures. In addition, advanced intraductal lithotripsy modalities, such as electrohydraulic lithotripsy and laser lithotripsy, can be delivered through cholangioscopes, allowing effective fragmentation of stones that are refractory to conventional ERCP‐based techniques. Recent advances in digital cholangioscopes have further improved image resolution, maneuverability, and irrigation control, broadening their clinical utility. Despite these technological improvements, POCS and POPS remain technically demanding, and their indications must be carefully selected according to ductal anatomy and the clinical scenario. In particular, the narrow diameter of the main pancreatic duct limits the application of POPS, restricting its use to specific situations, such as intraductal papillary mucinous neoplasms or indeterminate ductal strictures. Overall, the integration of cholangioscopy and intraductal lithotripsy into ERCP practice represents a significant advancement, enabling more precise diagnosis and expanding therapeutic options for complex pancreatobiliary disorders. This narrative review summarizes recent progress in diagnostic and therapeutic applications of cholangiopancreatography in pancreatobiliary disease.

## 1 Introduction

Despite advances in non‐invasive diagnostic modalities such as computed tomography (CT) and magnetic resonance imaging (MRI) and development of devices for stone fragmentation, there are still challenging cases in clinical practice [1]. Peroral cholangioscopy (POCS) and peroral pancreatoscopy (POPS) play important roles in addressing these issues. These techniques enable direct visualization and allow biopsy under direct vision for indeterminate strictures and treatment of difficult stones by stone fragmentation using electrohydraulic lithotripsy (EHL) or endoscopic laser lithotripsy (ELL). Recently, various types of cholangiopancreatoscopes, artificial intelligence (AI)‐assisted approaches, dedicated devices, and techniques such as antegrade procedures via endosonography‐created routes (ESCRs) have been reported. In this review, we summarize recent advances in diagnosis and treatment with cholangiopancreatoscopy.

## 2 Recent Advances in Cholangiopancreatoscopes

Since the first report of POCS in 1976 by Nakajima et al. [2], substantial technical and endoscopic advances have been achieved, particularly in recent years. POCS techniques can be broadly classified into three categories: mother–baby, direct, and single‐operator techniques.

In the mother–baby method, the latest POCS (CHF‐B290; Olympus Medical Systems, Tokyo, Japan) has the potential to improve diagnostic performance for biliary disease. POCS using this scope enables the use of texture and color image‐enhanced endoscopy (IEE) systems (EVIS X1; Olympus Medical Systems) [3]. According to a randomized controlled trial [4] and systematic review [5], texture and color image‐enhanced imaging (TXI) significantly improves ADR, polyp detection rate, and adenomas per patient compared with white light imaging, highlighting its potential to enhance the efficacy of colorectal cancer screening without impacting user experience or being affected by bowel preparation or withdrawal time. Therefore, TXI has clinical utility during gastrointestinal endoscopy. Tanisaka et al. [6] reported a case of biliary stricture in which TXI allowed clear visualization of the lesion, leading to a diagnosis of cholangiocarcinoma through optimization of mucosal surface structure, color, and brightness. Ishii et al. also suggested that TXI may be useful in the diagnosis of primary sclerosing cholangitis (SC) and IgG4‐related SC by enhancing the visualization of vascular structures [7, 8]. However, only a limited number of studies have reported its usefulness during POCS or POPS [6, 7, 8, 9]; therefore, further evaluative studies are warranted.

Direct POCS (D‐POCS) can be performed using an ultra‐slim gastroscope advanced with a monorail technique over a guidewire deployed in the bile duct or with balloon assistance [10]. Compared with digital cholangiopancreatoscopes, high‐quality images, including IEE systems, can be obtained, and because of the wider working channel, larger biopsy forceps can be used. However, D‐POCS can be technically challenging in cases of insufficient bile duct dilatation. In addition, stable scope positioning can also be challenging. Multi‐bending ultra‐slim scopes have been developed to address these limitations [11, 12, 13]. Lee et al. [12] conducted a retrospective analysis to evaluate the technical feasibility of POCS using multi‐bending ultra‐slim scopes with a free‐hand technique. Among 145 patients, multi‐bending ultra‐slim scopes could be inserted using the free‐hand technique in 133 patients (91.7%), and biopsy under D‐POCS guidance and therapeutic interventions such as stone fragmentation were successfully performed in 36 patients (94.7%, 36/38) and 65 patients (94.2%, 65/69), respectively.

Advances in single‐operator cholangiopancreatoscopes have also been observed, especially for disposable types [14, 15]. Table 1 summarizes currently available disposable cholangiopancreatoscopes. As no rigorous randomized controlled trials have directly compared cholangiopancreatoscopes, it is essential to understand the characteristics of each device and use them appropriately.

**TABLE 1:** Currently available disposable cholangiopancreatoscopes.^a^

Model	Company	Outer diameter, mm	Length of scope, mm	Diameter of working channel, mm	Bending capability	Extent of up/down angulation, degrees	Extent of right/left angulation, degrees
SpyGlass DS II	Boston Scientific	3.6	2140	1.2	Four‐way	90	90
eyeMAX 1.1	Micro‐Tech	3.2	2200	1.2	Four‐way	90	90
eyeMAX 2.0	Micro‐Tech	3.9	2200	2.0	Four‐way	90	90
mi‐see XO	Sungjin Medical	3.1	2000	1.18	Four‐way	160	120
ZISHORT	ZT Vision	3.1	2630	1.2	Four‐way	100	100
Briview	Briview Medical	3.8	2000	1.2	Four‐way	120	120
D‐Bile Xlink	Micro‐Tech	3.1	1980	1.2	Four‐way	130	130
Lan‐EP‐3516	Leinzett	3.5	2100	1.2	Four‐way	90	90
Scivita DCS	Scivita Medical	3.7/2.95	2100	2.0/1.2	Four‐way	90	90
VedScope	VedVision	3.6	2100	1.2	Four‐way	90	90

^a^ Specifications were obtained from manufacturer technical documents because detailed device parameters have not yet been uniformly reported in the literature.

## 3 Recent Advances in Diagnosis

**FIGURE 1:**
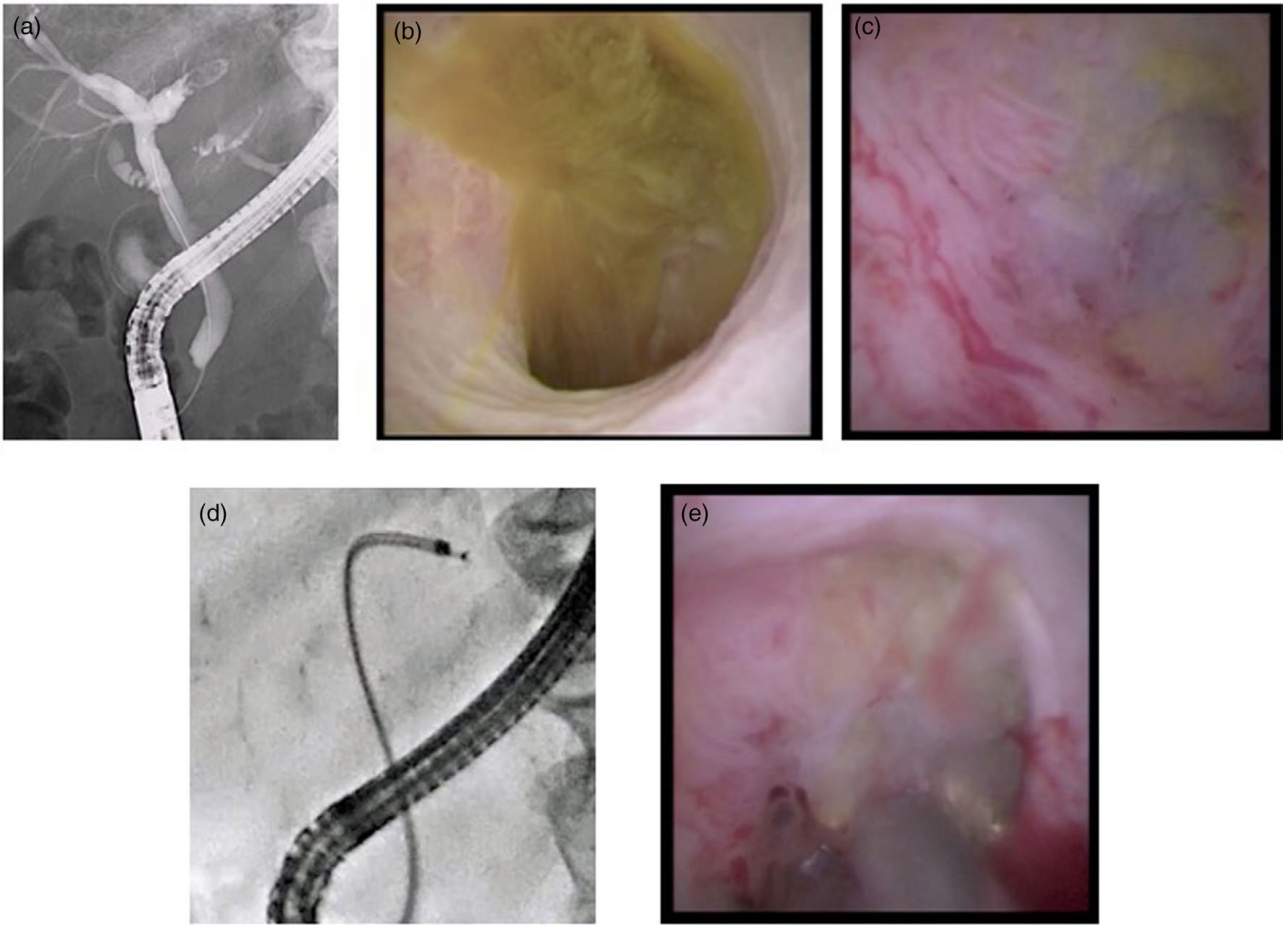
Cholangioscopy‐guided biopsy for intraductal mucinous neoplasm in the bile duct. (a) Cholangiography shows a defect in the left hepatic bile duct. (b) Mucin is observed. (c) After advancement of the cholangioscope into the confluence of B2 and B3, a tumor is observed. (d) Forceps biopsy at the confluence of B2 and B3 is challenging for fluoroscopy‐guided biopsy, but cholangioscopy‐guided biopsy is feasible. (e) Forceps biopsy is performed under direct visualization (fluoroscopic image).

The first‐line approaches for the diagnosis of biliary disease are brushing cytology or forceps biopsy under ERCP guidance. According to a meta‐analysis regarding brushing cytology and forceps biopsy under ERCP guidance [16], the diagnostic yield may not be sufficient in clinical practice. In contrast, POCS may have several advantages, such as providing direct ductal visualization and enabling targeted biopsy under visual guidance (Figure 1).

## 4 Recent Advances in Visual Findings

### 4.1 Biliary Disease

There are several proposed criteria for the differential diagnosis of malignant and benign biliary strictures. The Monaco classification is based on eight visual criteria [17], of which the presence of papillary projections (odds ratio [OR], 7.2; *p* = 0.025) and the presence of ulcers (OR, 10.3; *p* = 0.01) have been identified as strong factors associated with malignancy. In the recently reported Mendoza classification [18], the presence of tortuous and dilated vessels, irregular nodulations, raised intraductal lesions, irregular surface with or without ulcerations, and friability are considered suggestive of malignancy. Using these criteria, the diagnostic accuracy was 77%. In a recent meta‐analysis of the diagnostic yield of visual findings, overall sensitivity was 93% and specificity was 86%. There were no significant differences between digital and video cholangioscopy in terms of sensitivity (91% and 87%, respectively) or specificity (90% and 97%, respectively) [19].

Confocal laser endomicroscopy (CLE) is a recently introduced endoscopic imaging technique that enables “virtual biopsy” by performing in vivo histological evaluation in real time. Probe‐based CLE (pCLE; CholangioFlex, Cellvizio; Mauna Kea Technologies) can be advanced to the lesion under POCS or catheter guidance. Two pCLE classification systems have been proposed for evaluating indeterminate biliary strictures: the Miami and Paris classifications [20, 21]. According to a recent meta‐analysis of pCLE for indeterminate biliary strictures [22], sensitivity was 88% (95% CI, 0.84–0.91), and specificity was 79% (95% CI, 0.74–0.83). However, compared with the catheter‐based technique, pCLE under POCS guidance provides more reliable performance because the target lesion can be directly identified [23]. Accordingly, pCLE under POCS has been reported. Because previous studies have included both catheter‐based and POCS‐based techniques, the diagnostic yield attributable specifically to POCS‐guided pCLE remains unclear. Tanisaka et al. [23] prospectively assessed pCLE under POCS guidance in three cases, and achieved correct diagnoses in all patients. pCLE is only available in specialized tertiary centers; however, its widespread adoption remains limited by equipment availability, cost, and operator dependency [22]. Furthermore, standardized training protocols have not yet been fully established. A measurable learning curve for both image acquisition and interpretation, and structured training programs have been shown to improve diagnostic accuracy and interobserver agreement [24]. These factors should be considered when interpreting the clinical applicability of pCLE.

Artificial intelligence is now widely applied in upper and lower endoscopic fields. To date, AI has also been applied to cholangioscopy. Table 2 shows the diagnostic yield of AI‐assisted POCS for biliary disease [25, 26, 27, 28, 29, 30, 31, 32, 33, 34, 35]. According to previous studies, the sensitivity of conventional convolutional neural networks (CNNs) for malignant disease ranged from 81% to 99.7%, specificity ranged from 68.2% to 99.4%, and accuracy ranged from 83.9% to 99.3%. Also, a novel AI system employing a Vision Transformer architecture has recently been developed [34]. Although further evaluation is needed, AI systems may open a new avenue in clinical practice for diagnosing biliary disease.

**TABLE 2:** Diagnostic yield of artificial intelligence‐assisted peroral cholangioscopy for biliary disease.

Author	Year	Design	Model	No. of patients	Sensitivity, %	Specificity, %	Accuracy, %
Ribeiro et al. [24]	2021	Malignant vs. benign	CNN, Frame analysis	85	99.7	97.1	98.2
Pereira et al. [25]	2022	Malignant vs. benign	CNN, Frame analysis	85	99.3	99.4	99.3
Saraiva et al. [26]	2022	Malignant vs. benign	CNN, Frame analysis	85	94.7	92.1	94.9
Marya et al. [27]	2023	Malignant vs. benign	CNN, Real‐time analysis	122	93.3	88.2	90.6
Robles‐Medranda et al. [28]	2023	Malignant lesion detection	CNN, real‐time analysis	116	90.6	68.2	80.0
Zhang et al. [29]	2023	Malignant vs. benign	Data‐efficient image transformer, Frame analysis	94	95.6	89.0	92.3
Saraiva et al. [30]	2023	Malignant vs. benign	CNN, Frame analysis	129	83.5	82.4	AUC 0.92
Robles‐Medranda et al. [31]	2024	Malignant lesion detection	CNN, Real‐time analysis	90	97.7	75	96.7
Mascarenhas et al. [32]	2025	Malignant vs. benign	CNN, Frame analysis	164	91.7	94.4	92.9
Marya et al. [33]	2025	Malignant vs. benign	CNN, Frame analysis	99	81	95	91
Sato et al. [34]	2025	Malignant vs. benign	Vision Transformer architecture, Frame analysis	132	85.6	81.3	83.9

Abbreviations: AUC, area under the receiver‐operating characteristic curve; CNN, convolutional neural network.

### 4.2 Pancreatic Disease

**FIGURE 2:**
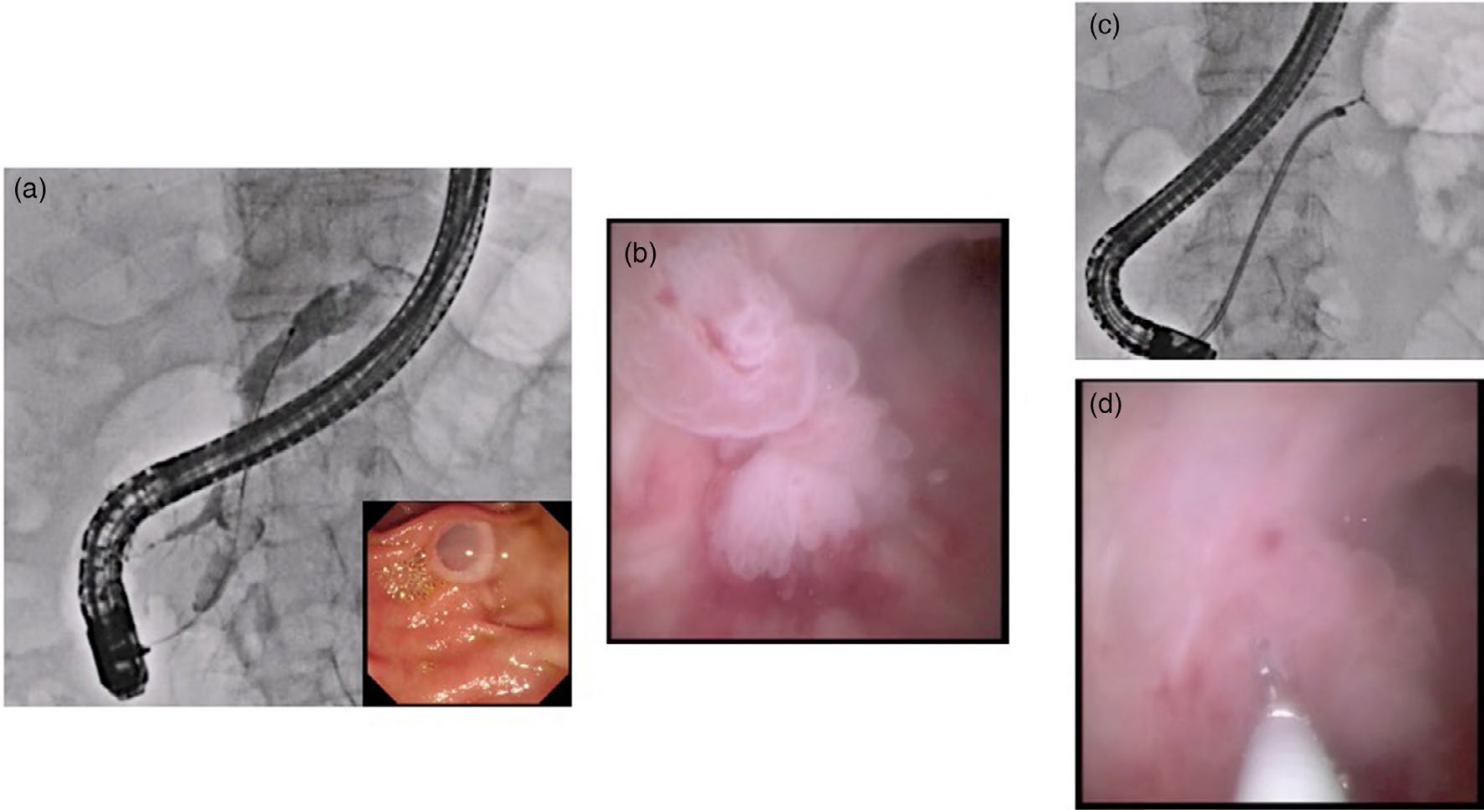
Pancreatoscopy‐guided biopsy for intraductal papillary mucinous neoplasm (IPMN). Pancreatography shows a defect in the main pancreatic duct of the pancreatic head, suspected to represent mucin (a). Although a mural nodule was not detected by non‐invasive imaging modalities, a mural nodule can be identified by pancreatoscopy (b). Biopsy is performed (c, d).

Compared with POCS, the indications for POPS are limited because the MPD is usually narrow. However, if POPS can be attempted, significant information of visual finding can be obtained. Typically, malignant lesions are characterized by papillary or villous projections, mucosal nodularity, irregular surfaces, abnormal tumor vessels, and friability, whereas benign strictures often show a smooth duct wall, concentric narrowing, and inflammatory changes [36, 37, 38, 39]. In clinical practice, POPS is indicated for the evaluation of MPD abnormalities such as intraductal papillary mucinous neoplasm (IPMN) or indeterminate MPD stricture (Figure 2), but not when the diameter of the MPD is less than 5 mm [36]. Despite recent advances in non‐invasive diagnostic modalities such as contrast‐enhanced multi‐detector computed tomography (MDCT), MRI, and endoscopic ultrasound (EUS), some patients may undergo pancreatic surgery for IPMNs or benign lesions that cannot be definitively diagnosed using imaging techniques alone [37]. In this setting, POPS offers a critical advantage by clarifying diagnoses unresolved by standard imaging and identifying lesions overlooked on cross‐sectional modalities in as many as 42% of patients [38]. According to a meta‐analysis of the diagnostic yield of POPS for IPMN [37], sensitivity rates ranged between 64% and 100%, specificity rates between 75% and 100%, and overall diagnostic accuracy between 87.5% and 100%. In addition, the capability of POPS to assess malignant or high‐grade dysplastic changes and to delineate lesion extent contributes to optimized surgical strategies and the avoidance of unwarranted procedures. Indeed, POPS findings altered the surgical approach in 13%–62% of patients. In addition, Tyberg et al. [39] reported that among 13 patients who underwent preoperative mapping biopsy using POPS, surgical procedures were modified in 62% of those with main pancreatic duct lesions. Of these, 31% required a more extensive resection, whereas in the remaining 31%, the extent of resection was reduced. Intraoperative POPS (IPO) can be also considered to determinate surgical margin. According to meta‐analysis including 5 studies with 142 patients [40], IOP detected additional lesions in 34% of patients and resulted in changes to the surgical plan in 34%, with no reported procedure‐related complications. Although these findings suggest that IOP may help guide surgical strategy, the available studies are small and heterogeneous, highlighting the need for large multicenter prospective studies.

## 5 Recent Advances in Obtaining Histological Tissue

According to a recent meta‐analysis [41], the pooled diagnostic sensitivity was 56.0% (95% CI, 48.8%–63.1%, *I* ^2^ = 83.0%) with brushing, 67.0% (95% CI, 60.2%–73.5%, *I* ^2^ = 72.5%) with biopsy, and 70.7% (95% CI, 64.1%–76.8%, *I* ^2^ = 42.7%) with brushing and biopsy for biliary disease. Because these techniques are performed under fluoroscopic guidance and direct visualization cannot be obtained, their diagnostic yield may be insufficient. In contrast, POCS‐guided biopsy has the advantage of being performed under direct visualization. In this section, given that EUS‐guided fine‐needle biopsy is established for pancreatic tumors, POCS‐guided biopsy is mainly focused.

POCS‐guided biopsy is indicated for obtaining histological evidence and diagnosing superficial tumor extension. According to a meta‐analysis of the diagnostic yield of POCS that included 876 patients [19]. Sekine et al. [42] compared the obtained sample size between digital single‐operator cholangioscopes (DSOCS) and fluoroscopic‐guided biopsy. Compared with DSOC‐guided biopsy, the obtained samples were significantly larger (0.9 mm^2^ vs. 1.77 m^2^, *p* < 0.001). Several efforts have recently been reported to overcome this limitation. A comparative study between the conventional biopsy device (Spy‐Bite, Boston Scientific) and Spy‐Bite MAX [43] reported that tissue sample size was larger in the Spy‐Bite MAX group (mean, 1.8 ± 1.6 mm^2^) than in the Spy‐Bite group (mean, 1.0 ± 0.9 mm^2^; *p* = 0.004). The use of a larger diameter cholangioscope (eyeMAX, 11Fr) has also been reported for this purpose because larger forceps biopsy device can be used. According to a prospective evaluation study [14], tissue sample size was larger in the eyeMAX group (2.96 ± 0.69 mm^2^) than in the SpyGlass DS II group (1.80 ± 1.61 mm^2^). The bite‐on‐bite biopsy technique has also been reported [44, 45, 46]. Although the results were negative, further evaluation is warranted because larger samples can be obtained [46].

**FIGURE 3:**
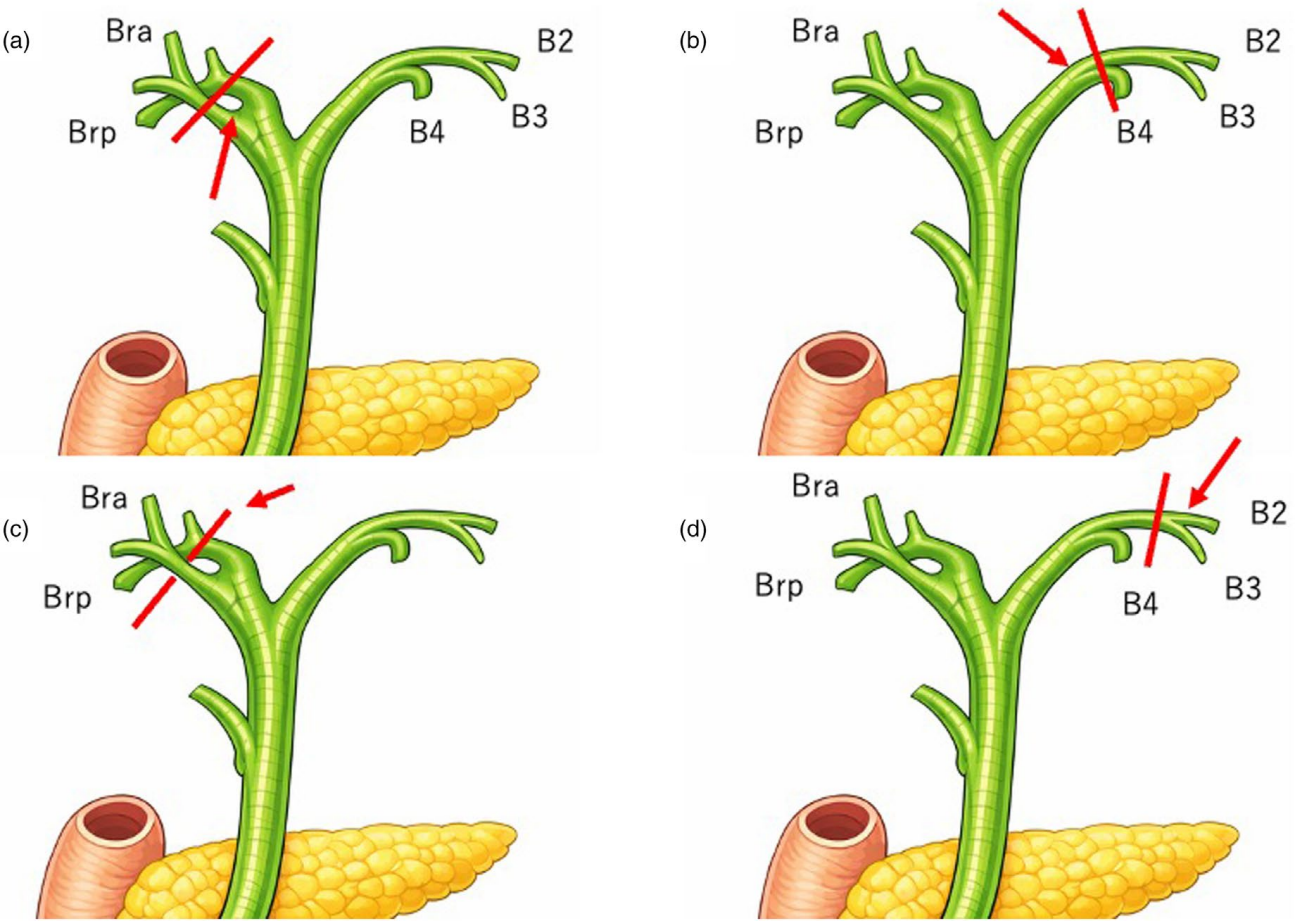
Transection lines and target biopsy sites. The transection lines are indicated by red lines, and the target biopsy sites are shown by red arrows. (a) In left hepatectomy with caudate lobectomy, a biopsy is obtained from the confluence of the right anterior and posterior sectoral ducts. (b) In right hepatectomy with caudate lobectomy, a biopsy is obtained from the confluence of B4 and B2 + 3. (c) In left trisectionectomy with caudate lobectomy, a biopsy is obtained from the apex of the right posterior sectoral duct (the portion crossing the portal vein). (d) In right trisectionectomy, a biopsy is obtained from B2 + 3.

POCS may also play a role in preoperative mapping biopsy [47, 48, 49], which can potentially alter surgical indication criteria (Figure 3). In fact, Tyberg et al. [40] reported that among 105 patients, mapping biopsies resulted in changes in the surgical plan in 32 cases (30%). Ogawa et al. [49] conducted a randomized crossover trial between POCS‐guided and fluoroscopic‐guided biopsy as a preoperative biopsy technique for cholangiocarcinoma. Among 28 patients, the success rate for approaching intrahepatic sites was significantly higher with POCS‐guided biopsy. The overall rates of site‐based successful biopsies were 78% (92/118) using POCS‐guided biopsy and 64% (76/118) using fluoroscopic‐guided biopsy (*p* = 0.031). Although stricture dilation is sometimes needed for cholangioscope insertion into the intrahepatic sites, POCS may play an important role in preoperative evaluation.

POPS‐guided biopsy also enables targeted tissue sampling under direct visualization and may improve the diagnostic yield for indeterminate pancreatic duct strictures. Previous studies have reported that the combination of visual impression and POPS‐guided biopsy provides high diagnostic accuracy for pancreatic duct neoplasms. According to previous study [50], diagnostic sensitivity, specificity, and accuracy for IPMN was 87%, 100%, and 92%, respectively. However, because of limited lumen compared with biliary tract, POPS‐guided biopsy might be challenging. Recently, 9Fr and 7.8Fr cholangiopancreatoscopes have been available [15, 51]. Their smaller diameter compared with conventional cholangiopancreatoscopes may facilitate ductal insertion. Further evaluation is needed.

## 6 Recent Advances in Treatment

POPS and POCS can be mainly used for stone management. As another application, several authors described POCS‐assisted cannulation [52, 53, 54, 55]. Liu et al. [52] reported initial experience of biliary cannulation using POCS. In their study, a cholangioscope fitted with a conical transparent cap was advanced toward the papilla and inserted into the orifice under direct visualization. The cap facilitated opening of the papillary folds and alignment with the bile duct axis, enabling direct entry into the common bile duct, occasionally with guidewire assistance. Technical success was obtained in all patients. Also, due to recent improvement of slim cholangioscope [55], this technique might be one of the options for challenging biliary cannulation cases, but a prospective evaluation study is needed.

### 6.1 Recent Advances in Stone Treatment

**FIGURE 4:**
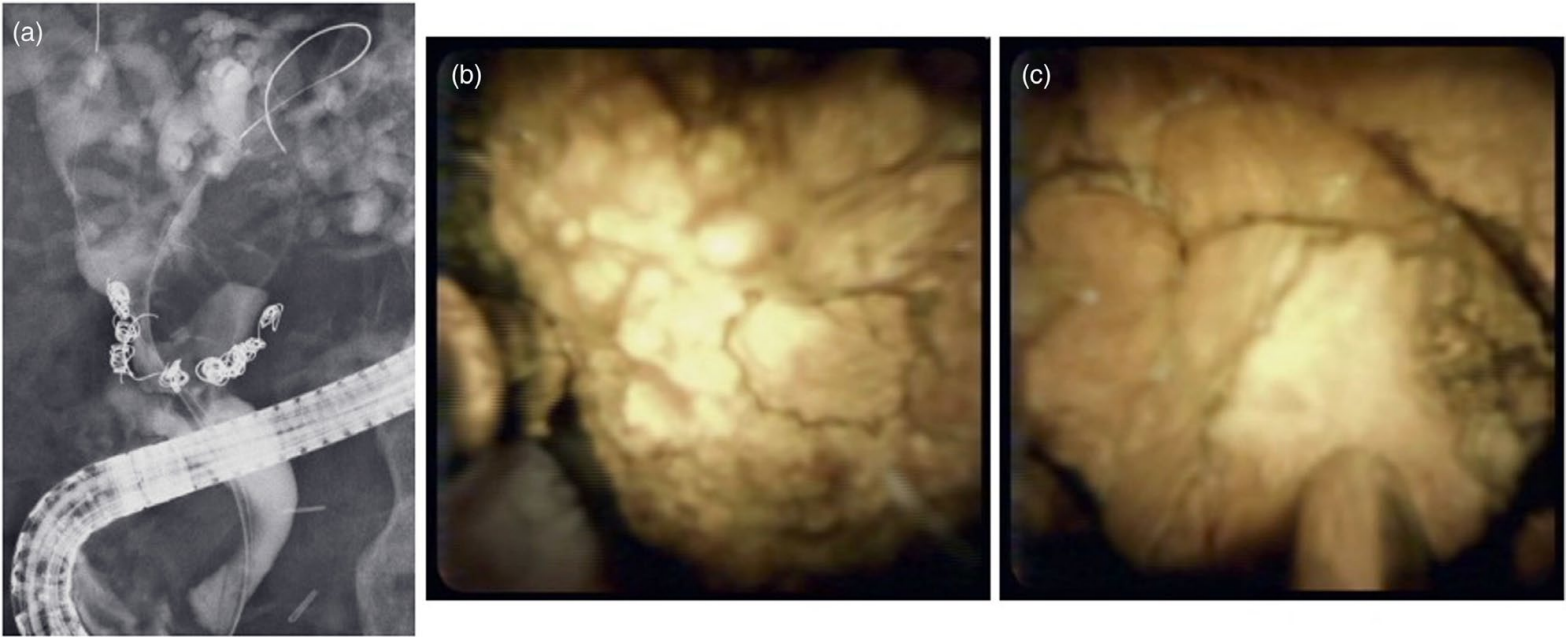
Electrohydraulic lithotripsy (EHL) for bile duct stone. (a) Large bile duct stones are observed. (b) Bile duct stones are identified on cholangioscopy. (c) EHL is successfully performed.

**FIGURE 5:**
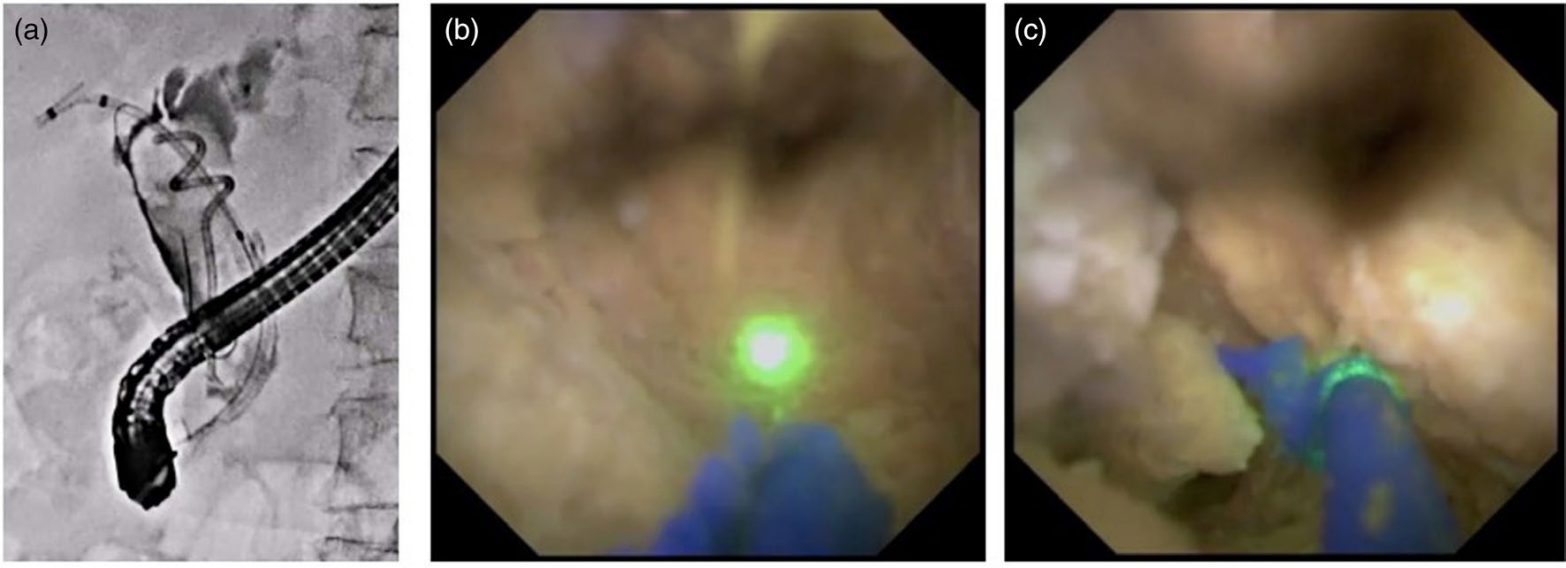
Laser lithotripsy (LL) for bile duct stone. (a) Large bile duct stones are observed. (b) Bile duct stones are identified on cholangioscopy. (c) LL is successfully performed.

Biliary stones can usually be treated using (mechanical) basket lithotripsy or balloon extraction under ERCP guidance, with a success rate of 85%–95% [56]. However, stone fragmentation is required in cases involving large stones (diameter > 1.5 cm), multiple stones, localization, and in the case of associated Mirizzi syndrome, which can be achieved by EHL or laser lithotripsy (LL) under POCS (Figures 4 and 5) [57, 58, 59, 60]. Table 3 shows the characteristics and relative advantages and disadvantages of EHL and LL [61, 62, 63, 64, 65]. In EHL, shock waves are generated by high‐voltage sparks and must be produced under water. In LL, shock waves are generated photomechanically and can be performed without water. Stone fragmentation efficacy may be higher in LL compared with EHL. In addition, energy controllability may be superior in LL because it is highly localized. Therefore, mucosal injury may be lower in LL compared with EHL. However, the cost of LL is high and the equipment occupies substantial space. Table 4 summarizes the findings of randomized controlled studies that have compared EHL/LL with other endoscopic techniques for bile duct stone removal [57, 58, 59, 60]. The initial complete stone clearance rate by EHL or LL ranged from 77.1% to 93.9%, which is significantly higher than with conventional endoscopic techniques, such as mechanical lithotripsy. Procedure times were generally longer with EHL and LL, but the rate of adverse events did not differ significantly. Bang et al. [60] conducted a randomized controlled trial comparing LL and endoscopic papillary large balloon dilation (EPLBD) and evaluated risk factors for technical success. According to multiple logistic regression analysis, the factors significantly associated with treatment success were the use of single‐operator cholangioscopic laser lithotripsy (SOC‐LL) (OR, 8.7; 95% CI, 1.3–59.3; *p* = 0.026), a stone/duct ratio 1 (OR, 28.8; 95% CI, 1.2–687.6; *p* = 0.038), and the absence of a tapered bile duct (OR, 26.9; 95% CI, 1.3–558.2; *p* = 0.034). In addition, their cost analysis found no significant difference in overall treatment cost between LL ($ 16,684) and EPLBD ($ 10,626) (*p* = 0.097). Therefore, they recommended that LL should be considered for patients with difficult bile duct stones, particularly when the stone/duct ratio is > 1.

**TABLE 3:** Comparison of characteristics between electrohydraulic and laser lithotripsy.

	EHL	LL
Mechanism	High‐voltage spark produces shock wave under water	Laser pulse produces photomechanical shock wave
Efficacy for stone fragmentation	High	Very high
Controllability of energy	Less focused	Highly localized
Probe characteristics	Flexible	Hard
Risk factors for complications	Mucosal injury due to shock wave	Penetration due to localized shock wave
Number of shock waves per session	Limited	Not limited (until probe failure)
Cost	Low	High
Size of machine	Compact	Large

Abbreviations: EHL, electrohydraulic lithotripsy; LL, laser lithotripsy.

**TABLE 4:** Summary of randomized controlled trials comparing electrohydraulic lithotripsy, laser lithotripsy, and other endoscopic techniques.

Authors	Year	Arm	Patients (n)	Number of stones (n)	Stone size (mm)	Initial success rate, % (n)	Overall technical success rate, % (n)	Procedure time (min)	Adverse events, % (n)
Franzini et al. [15]	2018	EHL	48	> 3, 31 1–3, 17	< 15, 9 15–20, 18 −20, 21	77.1 (37/48)	85.1 (40/47)	72.3	4.2 (2/48)
		EPLBD	50	> 3, 38 1–3, 12	< 15, 9 15–20, 19 −20, 22	72.0 (36/50)	95.4 (42/44)	47.1	12 (6/50)
		*p* value	—	0.216	0.995	> 0.05	0.1147	< 0.001	N.S.
Buxbaum et al. [51]	2018	LL	42	≥ 3, 9 2, 21 1, 12	19 (mean)	N/D	92.9 (39/42)	120.7	9.5 (4/42)
		Conventional	18	≥ 3, 2 2, 9 1, 7	18 (mean)	N/D	66.7 (12/18)	81.2	11.1 (2/18)
		*p* value	—	N.S.	N.S.	—	0.009	0.0014	N.S.
Angsuwatcharakon et al. [52]	2019	LL	16	2 (mean)	19.5 (mean)	100 (16/16)	N/A	66	6.3 (1/16)
		ML	16	2 (mean)	17.6 (mean)	62.5 (10/16)	81.3 (13/16)	83	12.5 (2/16)
		*p* value		> 0.99	0.28	< 0.01	—	0.23	0.76
Bang et al. [53]	2020	LL	33	3.7 (mean)	17.2 (mean)	93.9 (31/33)	100 (33/33)	39.2	9.1 (3/33)
		EPLBD	33	3.1 (mean)	15.3 (mean)	72.7 (24/33)	100 (33/33)	37.3	3.0 (1/33)
		*p* value	—	0.915	0.097	0.021	—	0.379	0.613

Abbreviations: EHL, electrohydraulic lithotripsy; EPLBD, endoscopic papillary large balloon dilation; LL, laser lithotripsy; ML, mechanical lithotripsy; N/A, not applicable; N/D, not described; N.S., not significant.

Several meta‐analyses comparing EHL and LL for difficult biliary stones have been reported [65, 66]. According to a meta‐analysis including 35 studies with 1762 patients [65], single‐session fragmentation success and duct clearance were higher with LL (82.9%, 95% CI, 75.0–88.7) compared with EHL (70.9%, 95% CI, 63.8–77.1) (*p* = 0.02), although overall fragmentation success did not differ significantly between EHL (90.1%, 95% CI, 82.1–94.8) and LL (92.8%, 95% CI, 88.2–95.7) (*p* = 0.36). Alexandrino et al. [67] evaluated factors influencing the performance of EHL or LL for difficult bile duct stones. Multivariate analysis showed that stone size independently predicted the need for multiple sessions (OR 1.146; 95% CI, 1.055–1.244; *p* = 0.001). ROC analysis identified 22 mm as the optimal cutoff for stone size (95% CI, 15.71–28.28; *p* < 0.001). EHL required significantly more probes than LL (2.0 vs. 1.02; *p* < 0.01). Therefore, although overall clinical efficacy may not differ substantially between EHL and LL for difficult stones and further randomized trials may be required, LL might be preferable for larger stones because it may reduce the number of treatment sessions and procedure time.

For pancreatic duct stones (PDs), extracorporeal shock wave lithotripsy (ESWL), with or without ERCP, including ERCP with pancreatic sphincterotomy, and stone removal using either a balloon or basket catheter, are the considerable approaches [68, 69]. According to a meta‐analysis regarding ESWL for PDs including 22 studies with 3868 patients [69], the pooled proportion of patients with complete ductal clearance was 69.8% (95% CI, 63.8–75.5), and the pooled proportion of complete absence of pain during follow‐up was 64.2% (95% CI, 57.5–70.6). Complete stone fragmentation was achieved in 86.3% (95% CI, 76.0–94.0). Post‐procedural pancreatitis and cholangitis occurred in 4.0% (95% CI, 2.5–5.8) and 0.5% (95% CI, 0.2–0.9), respectively. Although ESWL is undoubtedly useful for the treatment of PDs, in cases complicated by main pancreatic duct stricture, endoscopic treatment such as pancreatic duct stenting should be combined. However, a randomized trial has identified high cost as a disadvantage of combination therapy [70]. Current guidelines recommend that the management of PDs in patients with painful chronic pancreatitis should be tailored according to stone size, location, and radiopacity. The American Society for Gastrointestinal Endoscopy (ASGE) suggests that radiopaque stones larger than 5 mm located in the head, neck, or body of the pancreas can be treated with ERCP with or without POPS, or with ESWL alone. When adequate fragmentation is achieved after ESWL (defined as fragments < 2–3 mm) but spontaneous clearance does not occur, ERCP with or without pancreatoscopy is recommended to achieve stone clearance. For radiopaque stones smaller than 5 mm, radiolucent stones, or in patients with contraindications to ESWL, ERCP with or without POPS is suggested [71]. Similarly, the European Society of Gastrointestinal Endoscopy (ESGE) suggests considering pancreatoscopy‐guided lithotripsy when ESWL is not available or when stones remain unfragmented despite adequately performed ESWL [72].

**FIGURE 6:**
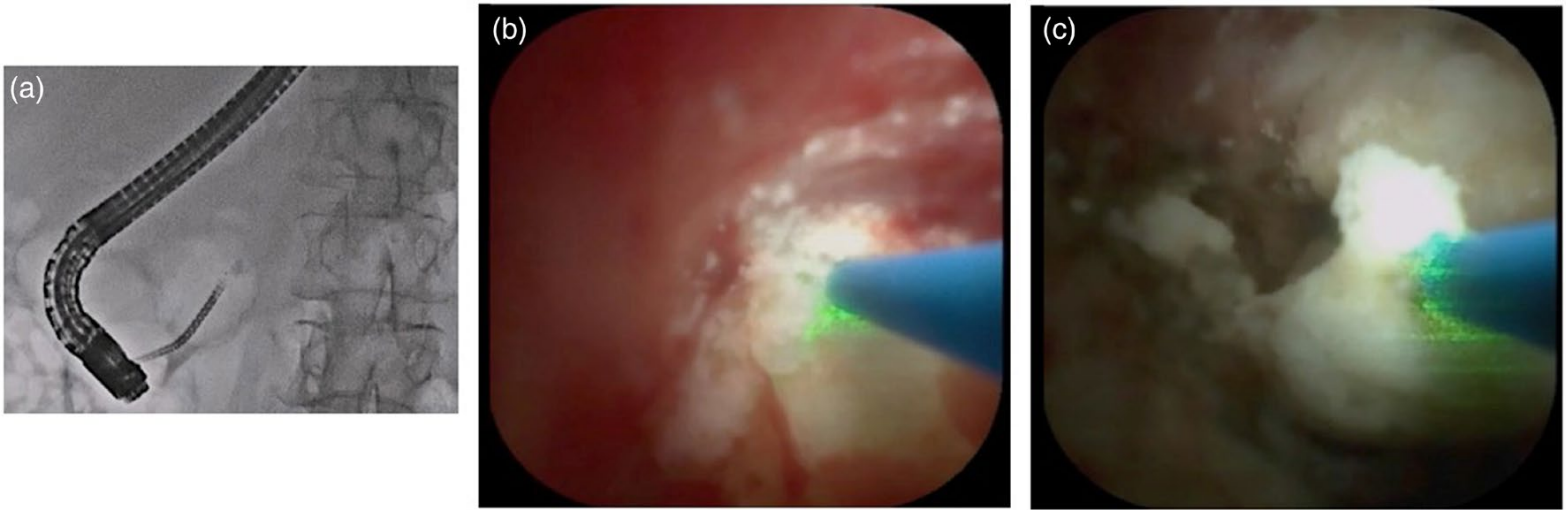
Laser lithotripsy (LL) for pancreatic duct stone. (a) Pancreatic duct stones are observed. (b) Pancreatic duct stones are identified on cholangioscopy. (c) LL is successfully performed.

EHL or LL for PDs are alternative techniques for failed ESWL. Following advances in pancreatoscope development, several recent studies have reported the usefulness of EHL or LL for PDs as a first‐line technique (Figure 6). According to a meta‐analysis comparing ESWL (*n* = 9251) and EHL/LL (238), including 9624 PDs cases [73], the pooled technical and clinical success rates of ESWL versus EHL/LL were similar. The rates of adverse events and post‐intervention pancreatitis for ESWL vs. EHL/LL were 10.1% (95% CI, 5.5%–17.6%) vs. 9.3% (95% CI, 4.1%–19.6%) (*p* = 0.87) and 4.3% (95% CI, 3.1%–5.9%) vs. 2.8% (95% CI, 1.3%–6.1%) (*p* = 0.32), respectively. A recent meta‐analysis that compared EHL and LL for PDs [74] reported a complete PDs clearance rate of 69% and a clinical success rate of 92% for EHL, with an adverse event (AE) rate of 9%. With LL, the complete clearance rate was 79%, the clinical success rate was 92%, and the AE rate was 8%. Further well‐designed, large‐scale randomized controlled trials are warranted to directly compare EHL‐POP and LL‐POP with ESWL, and to clarify whether POPS has the potential to serve as a first‐line therapy for PDs.

### 6.2 Recent Advances in Transluminal Treatment

EUS‐guided biliary drainage (EUS‐BD) is being increasingly performed for patients in whom ERCP is unsuccessful [75, 76, 77, 78], mainly to treat obstructive jaundice. Antegrade treatment via the EUS‐guided hepaticogastrostomy (HGS) or hepaticojejunostomy (HJS) route, such as bile duct stone removal or stricture treatment, has recently been reported [79, 80]. However, in cases involving large stones or the need to treat strictures, a cholangioscopic approach via the ESCR (the EUS‐HGS or HJS route) is needed [81, 82]. If a mature adhesion between the liver and the intestine has not formed at the ESCR, cholangioscope insertion carries a risk of bile leakage or perforation.

Table 5 lists the advantages and disadvantages of single‐step and second‐step antegrade treatments [83, 84, 85, 86, 87, 88, 89]. Single‐step antegrade treatment has several benefits, including low cost and shorter overall treatment duration; however, because an ESCR is not created, various devices (including a cholangioscope) cannot be used. In addition, transpapillary removal should be performed for bile duct stones. As a result, acute pancreatitis may occur as a complication.

**TABLE 5:** Advantages and disadvantages of single‐ and second‐step antegrade treatments.

	Advantages	Disadvantages
Single‐step antegrade treatment	Technically simple Low cost Shorter overall treatment duration	Risk of bile leakage or peritonitis Limited devices can be used Large stones and strictures cannot be treated
Second‐step antegrade treatment	Safe Various devices can be used, including POCS Allows transluminal stone removal	Time required for ESCR maturation High cost Longer overall treatment duration

Abbreviation: ESCR; endosonography‐created routes.

In second‐step antegrade treatment, various devices can be used after the formation of mature adhesion, including a cholangioscope. As transluminal stone removal can also be performed, pancreatitis may be avoided. However, because time is required for ESCR maturation and an additional stent is required, cost is high and a longer overall treatment duration is needed compared with single‐step antegrade treatment.

**FIGURE 7:**
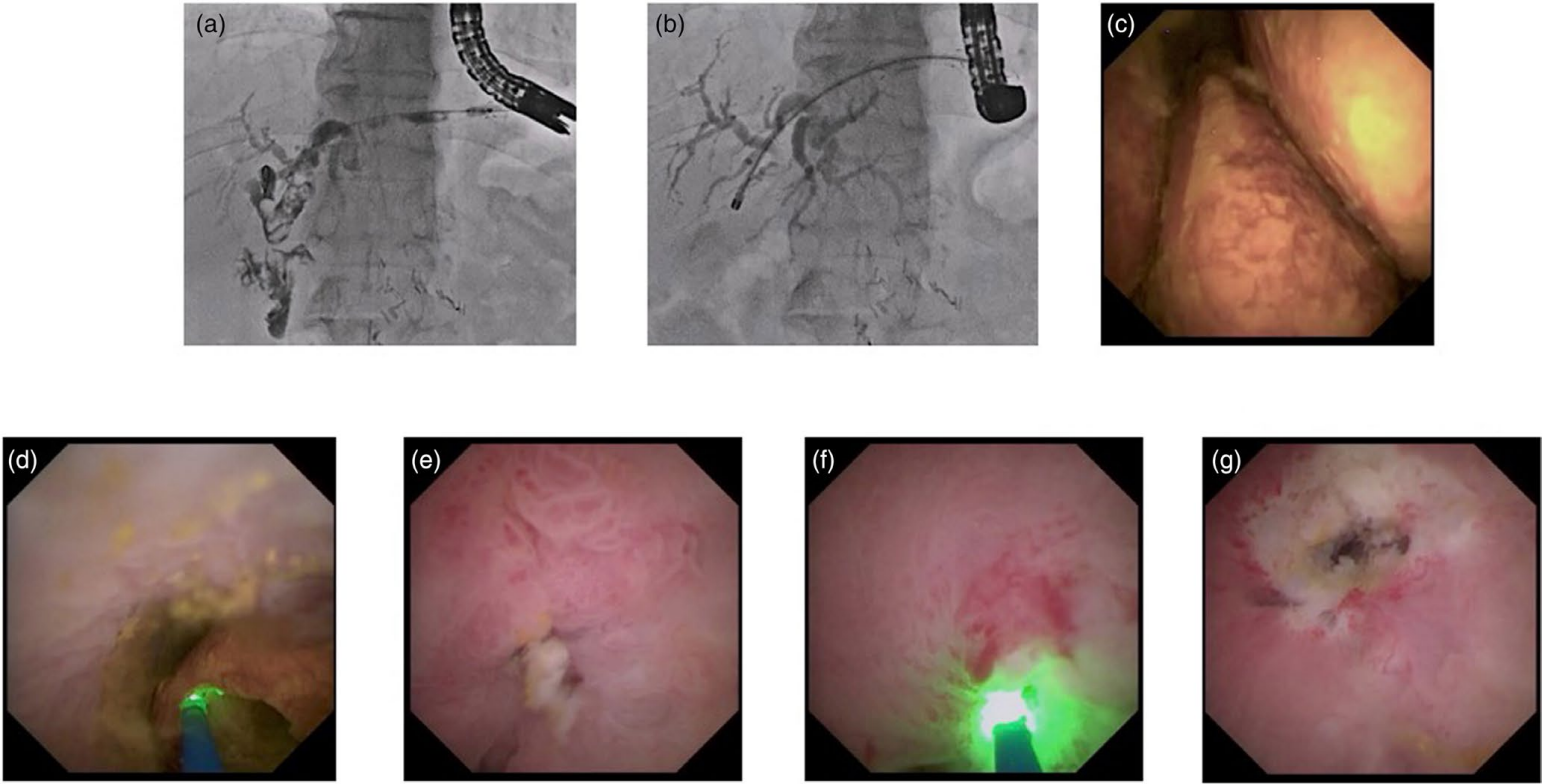
Transluminal cholangioscopy. (a) Cholangiography via the endoscopic ultrasound‐guided hepaticogastrostomy (EUS‐HGS) route shows bile duct stones and a hepaticojejunostomy stricture (HJS). (b) Cholangioscope insertion via the EUS‐HGS route is attempted. (c) Bile duct stones are identified. (d) Antegrade laser lithotripsy is performed. (e) HJS is also identified. (f) Antegrade laser ablation for HJS is attempted. (g) Stricture resolution is obtained.

Figure 7 shows a case of peroral transluminal cholangioscopy (PTLC) [88]. After successful EUS‐HGS, ESCR dilation is required to insert the cholangioscope, using a balloon catheter. If EUS‐HGS is performed using a self‐expandable metal stent (SEMS), cholangioscope insertion into the biliary system can be performed without ESCR dilation. After successful cholangioscope or pancreatoscope insertion, techniques such as EHL, LL, or stricture treatment using laser ablation can be attempted [83, 84, 85, 86, 87, 88, 89, 90, 91, 92, 93, 94, 95]. Mukai et al. [90] evaluated the outcome of antegrade intervention for benign biliary disease. In their study, second‐step antegrade treatment was attempted approximately 1 or 2 months later. Cholangioscopy‐assisted antegrade intervention was required in 19 cases (guidewire manipulation across the anastomotic stricture [*n* = 6], cholangioscopy‐guided lithotripsy using EHL [*n* = 13]). Magnetic compression anastomosis was performed in one case. The final clinical success rate of all EUS‐AIs was 91.9%.

**FIGURE 8:**
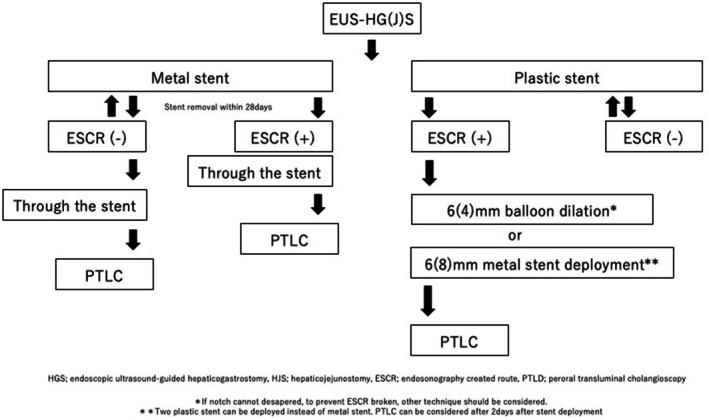
Flow chart of peroral transluminal cholangioscopy.

Figure 8 shows a proposed strategy for PTLC. If EUS‐HGS is performed using a metal stent, stent removal should be considered within approximately 28 days to prevent mucosal hyperplasia or difficulty in stent removal. If ESCR has not matured after stent removal, repeat stent deployment is performed. If ESCR is created, PTLC can be performed. Also, cholangioscope insertion can be performed through the proximal end of the metal stent. In comparison, if a plastic stent is deployed as the EUS‐HGS stent, ESCR dilation is needed to insert a cholangiopancreatoscope. Balloon dilation to 4 or 6 mm may be appropriate; however, this technique carries a risk of ESCR disruption. Although costly, PTLC should be deferred for several days until full stent expansion, and metal stent deployment may be safer. In the study of Hosmer et al. [89], ESCR formation was observed at 9, 34, 41, and 98 days after EUS‐HGS. In our previous study, stent removal was performed at 30.5 days (median; range, 28–38 days) after EUS‐HGS [85]. According to these previous studies, ESCR may form within approximately 28 days. However, because prospective evaluation of the time to ESCR formation has not been reported, further prospective studies are needed. On the contrary, the strategy of the transluminal pancreatic approach might be different. During EUS‐guided pancreatic duct drainage, to prevent pancreatic duct obstruction, a plastic stent might usually be selected. Therefore, the time to ESCR creation is still unclear. Using the peroral transluminal pancreatoscope (PTLP), stricture treatment, biopsy, and EHL/LL can be performed. However, there are no large‐scale studies of the PTLP although several case reports or series have been published [93, 94, 96, 97, 98, 99]. Further evaluation of this technique in a prospective setting is required.

As approach route for patients with inaccessible papilla, percutaneous or enteroscopic approach can be selected. Percutaneous transhepatic cholangioscopy (PTCS) using recent cholangioscope has emerged. A recent meta‐analysis including 12 studies (998 patients) demonstrated a higher clinical success rate with SpyGlass‐assisted PTCS compared with conventional PTCS (99% vs. 84%, *p* < 0.01), whereas technical success and adverse event rates were similar between the two approaches [100]. These findings suggest that SpyGlass‐assisted PTCS may improve clinical outcomes in challenging biliary access cases. Also, the development of balloon‐assisted enteroscopy (BAE) and thin cholangioscopes has enabled cholangioscopy‐guided diagnosis and therapy in patients with surgically altered anatomy. Several reports have demonstrated the feasibility of balloon enteroscopy‐assisted cholangioscopy for targeted biopsy or lithotripsy [101, 102, 103]. In a retrospective study comparing double‐balloon enteroscopy (DBE) and PTBD [103], complete stone clearance rates were similar between the two approaches (85.7% vs. 90.2%). However, DBE was associated with a significantly shorter hospital stay (6 vs. 21 days). These findings suggest that DBE provides outcomes comparable to PTBD and may represent a preferable first‐line treatment option when the anastomosis can be reached. Recent advances in cholangioscopy have markedly improved outcomes across various procedural approaches. Currently, the choice of approach is largely determined by patient characteristics, institutional resources, and the expertise of the endoscopist. However, large‐scale international multicenter randomized controlled trials are required to determine the optimal strategy.

## 7 Conclusion

In summary, the development of cholangiopancreatoscopes and associated techniques has expanded diagnostic and therapeutic options for pancreatobiliary disease. Further technological advances are expected to improve patient care. Appropriate understanding and structured training in their use are essential.

## Author Contributions

Takeshi Ogura wrote the paper. Takeshi Ogura, Nga Nguyen Trong, and Jayanta Samanta revised the work critically for important intellectual content, provided final approval of the version to be published, and agree to be accountable for all aspects of the work in ensuring that questions related to the accuracy or integrity of any part of the work are appropriately investigated and resolved. Nga Nguyen Trong and Jayanta Samanta also contributed proofreading English translation.

## Funding

The authors have nothing to report.

## Conflicts of Interest

The authors declare no conflicts of interest.

## References

[1] R. S. Tang , “Endoscopic Evaluation of Indeterminate Biliary Strictures: Cholangioscopy, Endoscopic Ultrasound, or Both?,” Digestive Endoscopy 36 (2024): 778–788. 38014445 10.1111/den.14733 PMC12136245

[2] M. Nakajima , Y. Asaka , K. Fukumoto , et al., “Peroral Cholangiopancreatoscopy (PCPS) Under Duodenoscopic Guidance,” American Journal of Gastroenterology 66 (1976): 241–247. 998588

[3] T. Sato , “TXI: Texture and Color Enhancement Imaging for Endoscopic Image Enhancement,” Journal of Healthcare Engineering 2021 (2021): 5518948. 33880168 10.1155/2021/5518948 PMC8049784

[4] N. Toyoshima , T. Sakamoto , K. Shinmura , et al., “The Efficacy of Texture and Color Enhancement Imaging Observation in the Detection of Colorectal Lesions: A Multicenter, Randomized Controlled Trial (deTXIon Study),” Gastroenterology 169 (2025): 337–345.e2. 40113100 10.1053/j.gastro.2025.03.007

[5] C. Judge , D. Law , G. Jones , S. Picardo , and K. Ragunath , “Texture and Color Enhanced Imaging in the Diagnosis of Colonic Neoplasia: A Systematic Review and Analysis,” JGH Open 9 (2025): e70191. 40832005 10.1002/jgh3.70191 PMC12358731

[6] Y. Tanisaka , M. Mizuide , A. Fujita , et al., “Usefulness of Texture and Color Enhancement Imaging in Peroral Cholangioscopy,” Endoscopy 55 (2023): E58–E59. 36179719 10.1055/a-1938-8173 PMC9829547

[7] T. Ishii , T. Kaneko , A. Murakami , et al., “Cholangioscopy in IgG4‐Related Sclerosing Cholangitis Using Texture and Color Enhancement Imaging and Red Dichromatic Imaging,” Endoscopy 55 (2023): E1019–E1020. 37647934 10.1055/a-2155-4853 PMC10468263

[8] Y. Tanisaka , M. Mizuide , and S. Ryozawa , “Usefulness of Texture and Color Enhancement Imaging in Peroral Pancreatoscopy,” Journal of Hepato‐Biliary‐Pancreatic Sciences 30 (2023): 1201–1203. 36734109 10.1002/jhbp.1312

[9] T. Ishii , A. Murakami , K. Sugimori , and S. Maeda , “Cholangioscopy for Primary Sclerosing Cholangitis With Biliary Strictures Using Texture and Color Enhancement Imaging and Red Dichromatic Imaging,” American Journal of Gastroenterology 120 (2025): 284–285. 39887125 10.14309/ajg.0000000000002878

[10] J. H. Moon , G. Terheggen , H. J. Choi , and H. Neuhaus , “Peroral Cholangioscopy: Diagnostic and Therapeutic Applications,” Gastroenterology 144 (2013): 276–282. 23127575 10.1053/j.gastro.2012.10.045

[11] T. Itoi , D. Nageshwar Reddy , A. Sofuni , et al., “Clinical Evaluation of a Prototype Multi‐Bending Peroral Direct Cholangioscope,” Digestive Endoscopy 26 (2014): 100–107. 23560942 10.1111/den.12082 PMC3933760

[12] Y. N. Lee , J. H. Moon , T. H. Lee , et al., “Efficacy and Safety of Direct Peroral Cholangioscopy Using a New Multibending Ultra‐Slim Endoscope for the Management of Biliary Diseases,” Journal of Hepato‐Biliary‐Pancreatic Sciences 29 (2022): 1292–1299. 35658104 10.1002/jhbp.1189

[13] W. M. Lee , J. H. Moon , Y. N. Lee , et al., “Utility of Direct Peroral Cholangioscopy Using a Multibending Ultraslim Endoscope for Difficult Common Bile Duct Stones,” Gut Liver 16 (2022): 599–605. 35000935 10.5009/gnl210355 PMC9289834

[14] T. Ogura , S. Ueno , A. Hakoda , et al., “Diagnostic Yield of a Novel 11‐Fr Digital Cholangioscope for Indeterminate Biliary Disease Using Macroscopic‐On‐Site Evaluation: Prospective Comparative Study,” Journal of Gastroenterology and Hepatology 40 (2025): 1307–1314. 39948712 10.1111/jgh.16907 PMC12062919

[15] T. Ogura , J. Nakamura , T. Kanadani , K. Bessho , and H. Nishikawa , “Endoscopic Laser Lithotripsy for an Impacted Pancreatic Duct Stone Using a Novel Ultra‐Slim Cholangiopancreatoscope via the Minor Papilla,” Endoscopy 57 (2025): E1155–E1156. 41101728 10.1055/a-2710-6280 PMC12530895

[16] U. Navaneethan , B. Njei , V. Lourdusamy , R. Konjeti , J. J. Vargo , and M. A. Parsi , “Comparative Effectiveness of Biliary Brush Cytology and Intraductal Biopsy for Detection of Malignant Biliary Strictures: A Systematic Review and Meta‐Analysis,” Gastrointestinal Endoscopy 81 (2015): 168–176. 25440678 10.1016/j.gie.2014.09.017 PMC4824293

[17] A. Sethi , A. Tyberg , A. Slivka , et al., “Digital Single‐Operator Cholangioscopy (DSOC) Improves Interobserver Agreement (IOA) and Accuracy for Evaluation of Indeterminate Biliary Strictures: The Monaco Classification,” Journal of Clinical Gastroenterology 56 (2022): e94–e97. 32040050 10.1097/MCG.0000000000001321

[18] M. Kahaleh , M. Gaidhane , H. M. Shahid , et al., “Digital Single‐Operator Cholangioscopy Interobserver Study Using a New Classification: The Mendoza Classification (With Video),” Gastrointestinal Endoscopy 95 (2022): 319–326. 34478737 10.1016/j.gie.2021.08.015

[19] S. Kulpatcharapong , R. Pittayanon , S. J. Kerr , and R. Rerknimitr , “Diagnostic Performance of Digital and Video Cholangioscopes in Patients With Suspected Malignant Biliary Strictures: A Systematic Review and Meta‐Analysis,” Surgical Endoscopy 36 (2022): 2827–2841. 34076761 10.1007/s00464-021-08571-2

[20] A. Meining , R. J. Shah , A. Slivka , et al., “Classification of Probe‐Based Confocal Laser Endomicroscopy Findings in Pancreatobiliary Stricture,” Endoscopy 44 (2012): 251–257. 22261749 10.1055/s-0031-1291545

[21] F. Caillol , B. Filoche , M. Gaidhane , and M. Kahaleh , “Refined Probe‐Based Confocal Laser Endomicroscopy Classification for Biliary Stricture: The Paris Classification,” Digestive Diseases and Sciences 58 (2013): 1784–1789. 23314855 10.1007/s10620-012-2533-5

[22] J. Mi , X. Han , R. Wang , R. Ma , and D. Zhao , “Diagnostic Accuracy of Probe‐Based Confocal Laser Endomicroscopy and Tissue Sampling by Endoscopic Retrograde Cholangiopancreatography in Indeterminate Biliary Strictures: A Meta‐Analysis,” Scientific Reports 12 (2022): 7257. 35508585 10.1038/s41598-022-11385-4 PMC9068817

[23] Y. Tanisaka , S. Ryozawa , M. Mizuide , et al., “Prospective Assessment of Probe‐Based Confocal Laser Endomicroscopy Under Direct Cholangioscopic Visualization for Biliary Strictures That Could Not Be Definitively Diagnosed Using Endoscopic Retrograde Cholangiopancreatography (With Video),” DEN Open 5 (2024): e70007. 39328351 10.1002/deo2.70007 PMC11424493

[24] M. Ayyad , D. Gala , M. Albandak , et al., “Probe‐Based Confocal Laser Endomicroscopy: Progress, Challenges, and Emerging Applications,” Surgical Endoscopy 39 (2025): 7958–7972. 41184675 10.1007/s00464-025-12297-w PMC12708787

[25] T. Ribeiro , M. M. Saraiva , J. Afonso , et al., “Automatic Identification of Papillary Projections in Indeterminate Biliary Strictures Using Digital Single‐Operator Cholangioscopy,” Clinical and Translational Gastroenterology 12 (2021): e00418. 34704969 10.14309/ctg.0000000000000418 PMC8553239

[26] P. Pereira , M. Mascarenhas , T. Ribeiro , et al., “Automatic Detection of Tumor Vessels in Indeterminate Biliary Strictures in Digital Single‐Operator Cholangioscopy,” Endoscopy International Open 10 (2022): E262–E268. 35295246 10.1055/a-1723-3369 PMC8920599

[27] M. M. Saraiva , T. Ribeiro , J. P. S. Ferreira , et al., “Artificial Intelligence for Automatic Diagnosis of Biliary Stricture Malignancy Status in Single‐Operator Cholangioscopy: A Pilot Study,” Gastrointestinal Endoscopy 95 (2022): 339–348. 34508767 10.1016/j.gie.2021.08.027

[28] N. B. Marya , P. D. Powers , B. T. Petersen , et al., “Identification of Patients With Malignant Biliary Strictures Using a Cholangioscopy‐Based Deep Learning Artificial Intelligence (With Video),” Gastrointestinal Endoscopy 97 (2023): 268–278. 36007584 10.1016/j.gie.2022.08.021

[29] C. Robles‐Medranda , J. Baquerizo‐Burgos , J. Alcivar‐Vasquez , et al., “Artificial Intelligence for Diagnosing Neoplasia on Digital Cholangioscopy: Development and Multicenter Validation of a Convolutional Neural Network Model,” Endoscopy 55 (2023): 719–727. 36781156 10.1055/a-2034-3803 PMC10374349

[30] X. Zhang , D. Tang , J. D. Zhou , et al., “A Real‐Time Interpretable Artificial Intelligence Model for the Cholangioscopic Diagnosis of Malignant Biliary Stricture (With Videos),” Gastrointestinal Endoscopy 98 (2023): 199–210. 36849057 10.1016/j.gie.2023.02.026

[31] M. M. Saraiva , T. Ribeiro , M. González‐Haba , et al., “Deep Learning for Automatic Diagnosis and Morphologic Characterization of Malignant Biliary Strictures Using Digital Cholangioscopy: A Multicentric Study,” Cancers 15 (2023): 4827. 37835521 10.3390/cancers15194827 PMC10571941

[32] C. Robles‐Medranda , J. Baquerizo‐Burgos , M. Puga‐Tejada , et al., “Cholangioscopy‐Based Convoluted Neuronal Network vs. Confocal Laser Endomicroscopy in Identification of Neoplastic Biliary Strictures,” Endoscopy International Open 12 (2024): E1118–E1126. 39398445 10.1055/a-2404-5699 PMC11466527

[33] M. Mascarenhas , M. J. Almeida , M. González‐Haba , et al., “Artificial Intelligence for Automatic Diagnosis and Pleomorphic Morphological Characterization of Malignant Biliary Strictures Using Digital Cholangioscopy,” Scientific Reports 15 (2025): 5447. 39952950 10.1038/s41598-025-87279-y PMC11828993

[34] N. B. Marya , P. D. Powers , J. P. AbiMansour , et al., “Multicenter Validation of a Cholangioscopy Artificial Intelligence System for the Evaluation of Biliary Tract Disease,” Endoscopy 58 (2025): 789, 10.1055/a-2650-0789. 40618756

[35] R. Sato , K. Matsumoto , M. Tomiya , et al., “Vendor‐Agnostic Vision Transformer‐Based Artificial Intelligence for Peroral Cholangioscopy: Diagnostic Performance in Biliary Strictures Compared With Convolutional Neural Networks and Endoscopists,” Digestive Endoscopy 37 (2025): 1315–1322, 10.1111/den.70028. 40903880 PMC12702826

[36] R. J. Shah , “Innovations in Intraductal Endoscopy: Cholangioscopy and Pancreatoscopy,” Gastrointestinal Endoscopy Clinics of North America 25 (2015): 779–792. 26431604 10.1016/j.giec.2015.06.012

[37] D. M. de Jong , P. M. C. Stassen , B. Groot Koerkamp , et al., “The Role of Pancreatoscopy in the Diagnostic Work‐Up of Intraductal Papillary Mucinous Neoplasms: A Systematic Review and Meta‐Analysis,” Endoscopy 55 (2023): 25–35. 35668651 10.1055/a-1869-0180 PMC9767751

[38] A. J. Trindade , P. C. Benias , P. Kurupathi , et al., “Digital Pancreatoscopy in the Evaluation of Main Duct Intraductal Papillary Mucinous Neoplasm: A Multicenter Study,” Endoscopy 50 (2018): 1095–1098. 29698989 10.1055/a-0596-7374

[39] A. Tyberg , I. Raijman , A. Siddiqui , et al., “Digital Pancreaticocholangioscopy for Mapping of Pancreaticobiliary Neoplasia: Can we Alter the Surgical Resection Margin?,” Journal of Clinical Gastroenterology 53 (2019): 71–75. 29517713 10.1097/MCG.0000000000001008

[40] D. Ciprani , A. Frampton , H. Amar , K. Oppong , S. Pandanaboyana , and S. Aroori , “The Role of Intraoperative Pancreatoscopy in the Surgical Management of Intraductal Papillary Mucinous Neoplasms of the Pancreas: A Systematic Scoping Review,” Surgical Endoscopy 37 (2023): 9043–9051. 37907657 10.1007/s00464-023-10518-8

[41] S. B. Yoon , S. H. Moon , S. W. Ko , H. Lim , H. S. Kang , and J. H. Kim , “Brush Cytology, Forceps Biopsy, or Endoscopic Ultrasound‐Guided Sampling for Diagnosis of Bile Duct Cancer: A Meta‐Analysis,” Digestive Diseases and Sciences 67 (2022): 3284–3297. 34263382 10.1007/s10620-021-07138-4

[42] K. Sekine , I. Yasuda , S. Doi , et al., “Peroral Cholangioscopy‐Guided Targeted Biopsy Versus Conventional Endoscopic Transpapillary Forceps Biopsy for Biliary Stricture With Suspected Bile Duct Cancer,” Journal of Clinical Medicine 11 (2022): 289. 35053987 10.3390/jcm11020289 PMC8779099

[43] T. Ogura , Y. Hirose , S. Ueno , et al., “Prospective Registration Study of Diagnostic Yield and Sample Size in Forceps Biopsy Using a Novel Device Under Digital Cholangioscopy Guidance With Macroscopic On‐Site Evaluation,” Journal of Hepato‐Biliary‐Pancreatic Sciences 30 (2023): 686–692. 36196526 10.1002/jhbp.1247

[44] T. Ogawa , Y. Kanno , S. Koshita , et al., “Dose Performing Multiple Biopsy Strokes From the Same Site Improve Specimen Adequacy in Cholangioscopy‐Guided Mapping Biopsy for Extrahepatic Cholangiocarcinoma?,” JGH Open 9 (2025): e70246. 40771700 10.1002/jgh3.70246 PMC12326186

[45] D. M. de Jong , P. J. F. de Jonge , P. M. C. Stassen , et al., “The Value of Cholangioscopy‐Guided Bite‐On‐Bite (−On Bite) Biopsies in Indeterminate Biliary Duct Strictures,” Endoscopy 57 (2025): 1220–1229. 40409293 10.1055/a-2619-6803 PMC12566887

[46] T. Ogura , J. Nakamura , T. Kanadani , K. Bessho , and H. Nishikawa , “A Modified Tetra‐Bite‐On‐Bite Biopsy Technique Under Cholangioscopic Guidance for Biliary Disease,” Endoscopy 57 (2025): E1197–E1198. 41187781 10.1055/a-2723-1724 PMC12585899

[47] T. Ogawa , K. Ito , S. Koshita , et al., “Usefulness of Cholangioscopic‐Guided Mapping Biopsy Using SpyGlass DS for Preoperative Evaluation of Extrahepatic Cholangiocarcinoma: A Pilot Study,” Endoscopy International Open 6 (2018): E199–E204. 29399618 10.1055/s-0043-117949 PMC5794453

[48] Y. Kanno , S. Koshita , T. Ogawa , et al., “Peroral Cholangioscopy by SpyGlass DS Versus CHF‐B260 for Evaluation of the Lateral Spread of Extrahepatic Cholangiocarcinoma,” Endoscopy International Open 6 (2018): E1349–E1354. 30410956 10.1055/a-0743-5283 PMC6221821

[49] T. Ogawa , Y. Kanno , S. Koshita , et al., “Cholangioscopy‐ Versus Fluoroscopy‐Guided Transpapillary Mapping Biopsy for Preoperative Evaluation of Extrahepatic Cholangiocarcinoma: A Prospective Randomized Crossover Study,” Surgical Endoscopy 35 (2021): 6481–6488. 33141278 10.1007/s00464-020-08141-y

[50] I. I. El Hajj , B. C. Brauer , S. Wani , N. Fukami , A. R. Attwell , and R. J. Shah , “Role of Per‐Oral Pancreatoscopy in the Evaluation of Suspected Pancreatic Duct Neoplasia: A 13‐Year U.S. Single‐Center Experience,” Gastrointestinal Endoscopy 85 (2017): 737–745. 27473181 10.1016/j.gie.2016.07.040

[51] H. Miwa , K. Sugimori , K. Irie , et al., “Feasibility of Novel Slim Peroral Cholangiopancreatoscopy for the Diagnosis of Pancreatobiliary Disease,” DEN Open 6 (2025): e70152. 40458535 10.1002/deo2.70152 PMC12128889

[52] W. H. Liu , X. Y. Huang , X. Hu , et al., “Initial Experience of Visualized Biliary Cannulation During ERCP,” Endoscopy 55 (2023): 1037–1042. 37339664 10.1055/a-2113-8952

[53] W. H. Liu , X. Y. Huang , R. Y. Zhang , R. Huang , S. Y. Qin , and X. G. Liu , “From Darkness to Brightness: The Cholangioscopy‐Guided Selective Biliary Cannulation With the Help of Transparent Cap During ERCP,” Endoscopy 55 (2023): E320–E321. 36513111 10.1055/a-1981-2503 PMC9833945

[54] S. S. Hu , X. G. Liu , J. Hou , and W. H. Liu , “The Guidewire‐Guided Endoscopic Retrograde Direct Cholangioscopy Facilitating the Difficult Biliary Cannulation of Small Papilla,” Endoscopy 57 (2025): E653–E655. 40570916 10.1055/a-2602-3230 PMC12202116

[55] Y. Tanisaka , M. Mizuide , and S. Ryozawa , “Successful Cholangioscopy‐Guided Cannulation Using a Novel Slim Cholangioscope,” Journal of Hepatobiliary Pancreatic Sciences 31 (2024): e22–e24. 38282574 10.1002/jhbp.1416

[56] T. Ogura and K. Higuchi , “A Review of Treatment Options for Bile Duct Stones,” Expert Review of Gastroenterology & Hepatology 10 (2016): 1271–1278. 27410721 10.1080/17474124.2016.1212658

[57] T. Franzini , R. N. Moura , P. Bonifácio , et al., “Complex Biliary Stones Management: Cholangioscopy Versus Papillary Large Balloon Dilation ‐ a Randomized Controlled Trial,” Endoscopy International Open 6 (2018): E131–E138. 29399609 10.1055/s-0043-122493 PMC5794432

[58] J. Buxbaum , A. Sahakian , C. Ko , et al., “Randomized Trial of Cholangioscopy‐Guided Laser Lithotripsy Versus Conventional Therapy for Large Bile Duct Stones (With Videos),” Gastrointestinal Endoscopy 87 (2018): 1050–1060. 28866457 10.1016/j.gie.2017.08.021

[59] P. Angsuwatcharakon , S. Kulpatcharapong , W. Ridtitid , et al., “Digital Cholangioscopy‐Guided Laser Versus Mechanical Lithotripsy for Large Bile Duct Stone Removal After Failed Papillary Large‐Balloon Dilation: A Randomized Study,” Endoscopy 51 (2019): 1066–1073. 30786315 10.1055/a-0848-8373

[60] J. Y. Bang , B. Sutton , U. Navaneethan , R. Hawes , and S. Varadarajulu , “Efficacy of Single‐Operator Cholangioscopy‐Guided Lithotripsy Compared With Large Balloon Sphincteroplasty in Management of Difficult Bile Duct Stones in a Randomized Trial,” Clinical Gastroenterology and Hepatology 18 (2020): 2349–2356.e3. 32057976 10.1016/j.cgh.2020.02.003

[61] D. H. Birkett , J. S. Lamont , J. C. O'Keane , and R. K. Babayan , “Comparison of a Pulsed Dye Laser and Electrohydraulic Lithotripsy on Porcine Gallbladder and Common Bile Duct In Vitro,” Lasers in Surgery and Medicine 12 (1992): 210–214. 1349415 10.1002/lsm.1900120216

[62] E. Troncone , M. Mossa , P. De Vico , G. Monteleone , and G. Del Vecchio Blanco , “Difficult Biliary Stones: A Comprehensive Review of New and Old Lithotripsy Techniques,” Medicina 58 (2022): 120. 35056428 10.3390/medicina58010120 PMC8779004

[63] Y. Peng , M. Liu , S. Ming , et al., “Safety of a Novel Thulium Fiber Laser for Lithotripsy: An In Vitro Study on the Thermal Effect and Its Impact Factor,” Journal of Endourology 34 (2020): 88–92. 31608659 10.1089/end.2019.0426

[64] J. V. Veld , N. C. M. van Huijgevoort , M. A. Boermeester , et al., “A Systematic Review of Advanced Endoscopy‐Assisted Lithotripsy for Retained Biliary Tract Stones: Laser, Electrohydraulic or Extracorporeal Shock Wave,” Endoscopy 50 (2018): 896–909. 29991072 10.1055/a-0637-8806

[65] T. R. McCarty , R. Gulati , and T. Rustagi , “Efficacy and Safety of Peroral Cholangioscopy With Intraductal Lithotripsy for Difficult Biliary Stones: A Systematic Review and Meta‐Analysis,” Endoscopy 53 (2021): 110–122. 32544959 10.1055/a-1200-8064

[66] A. C. Amaral , W. K. Hussain , and S. Han , “Cholangioscopy‐Guided Electrohydraulic Lithotripsy Versus Laser Lithotripsy for the Treatment of Choledocholithiasis: A Systematic Review,” Scandinavian Journal of Gastroenterology 58 (2023): 1213–1220. 37203215 10.1080/00365521.2023.2214657

[67] G. Alexandrino , L. Lopes , J. Fernandes , et al., “Factors Influencing Performance of Cholangioscopy‐Guided Lithotripsy Including Available Different Technologies: A Prospective Multicenter Study With 94 Patients,” Digestive Diseases and Sciences 67 (2022): 4195–4203. 34811629 10.1007/s10620-021-07305-7

[68] K. Ito , K. Takuma , N. Okano , et al., “Current Status and Future Perspectives for Endoscopic Treatment of Local Complications in Chronic Pancreatitis,” Digestive Endoscopy 37 (2025): 219–235. 39364545 10.1111/den.14926 PMC11884972

[69] N. C. M. van Huijgevoort , J. V. Veld , P. Fockens , et al., “Success of Extracorporeal Shock Wave Lithotripsy and ERCP in Symptomatic Pancreatic Duct Stones: A Systematic Review and Meta‐Analysis,” Endoscopy International Open 8 (2020): E1070–E1085. 32743061 10.1055/a-1171-1322 PMC7373664

[70] J. M. Dumonceau , G. Costamagna , A. Tringali , et al., “Treatment for Painful Calcified Chronic Pancreatitis: Extracorporeal Shock Wave Lithotripsy Versus Endoscopic Treatment: A Randomised Controlled Trial,” Gut 56 (2007): 545–552. 17047101 10.1136/gut.2006.096883 PMC1856858

[71] S. G. Sheth , J. D. Machicado , J. M. Chalhoub , et al., “American Society for Gastrointestinal Endoscopy Guideline on the Role of Endoscopy in the Management of Chronic Pancreatitis: Summary and Recommendations,” Gastrointestinal Endoscopy 100 (2024): 584–594. 39115496 10.1016/j.gie.2024.05.016

[72] J. M. Dumonceau , M. Delhaye , A. Tringali , et al., “Endoscopic Treatment of Chronic Pancreatitis: European Society of Gastrointestinal Endoscopy (ESGE) Guideline – Updated August 2018,” Endoscopy 51 (2019): 179–193. 30654394 10.1055/a-0822-0832

[73] N. Siranart , L. Kozai , D. M. Simadibrata , et al., “Per‐Oral Pancreatoscopy‐Guided Lithotripsy Versus Extracorporeal Shock Wave Lithotripsy in Pancreatic Stone: A Meta‐Analysis,” Digestive Diseases and Sciences 70 (2025): 2506–2520. 40175795 10.1007/s10620-025-08952-w

[74] P. Huang , H. Khizar , W. Song , and J. Yang , “Pancreatoscopy‐Guided Lithotripsy for Pancreatic Duct Stones: A Systematic Review and Meta‐Analysis,” Turkish Journal of Gastroenterology 35 (2024): 811–821. 10.5152/tjg.2024.24110 PMC11562744 39548977

[75] T. Khoury , W. Sbeit , F. Fumex , et al., “Endoscopic Ultrasound‐ Versus ERCP‐Guided Primary Drainage of Inoperable Malignant Distal Biliary Obstruction: Systematic Review and Meta‐Analysis of Randomized Controlled Trials,” Endoscopy 56 (2024): 955–963. 38843824 10.1055/a-2340-0697

[76] E. C. Barbosa , P. A. D. E. Santo , S. Baraldo , A. L. Nau , and G. C. Meine , “EUS‐ Versus ERCP‐Guided Biliary Drainage for Malignant Biliary Obstruction: A Systematic Review and Meta‐Analysis of Randomized Controlled Trials,” Gastrointestinal Endoscopy 100 (2024): 395–405. 38648989 10.1016/j.gie.2024.04.019

[77] V. Moond , P. Loganathan , B. Koyani , et al., “Efficacy and Safety of EUS‐Guided Hepatogastrostomy: A Systematic Review and Meta‐Analysis,” Endoscopic Ultrasound 13 (2024): 171–182. 39318645 10.1097/eus.0000000000000055 PMC11419430

[78] K. Nagai , S. Mukai , M. Abe , et al., “Long‐Term Outcomes After EUS‐Guided Antegrade Intervention for Benign Bilioenteric Anastomotic Stricture,” Gastrointestinal Endoscopy 99 (2024): 50–60. 37562548 10.1016/j.gie.2023.07.052

[79] T. Iwashita , S. Uemura , R. Tezuka , A. Senju , I. Yasuda , and M. Shimizu , “Current Status of Endoscopic Ultrasound‐Guided Antegrade Intervention for Biliary Diseases in Patients With Surgically Altered Anatomy,” Digestive Endoscopy 35 (2023): 264–274. 35763410 10.1111/den.14393

[80] G. Dell'Anna , T. Ogura , G. Vanella , et al., “Endoscopic Ultrasound Guided Biliary Interventions,” Best Practice & Research. Clinical Gastroenterology 3 (2022): 60–61. 10.1016/j.bpg.2022.101810 36577530

[81] H. Isayama , Y. Nakai , K. Matsuda , et al., “Proposal of Classification and Terminology of Interventional Endoscopic Ultrasonography/Endosonography,” Digestive Endoscopy 37 (2025): 5–17. 39364550 10.1111/den.14927

[82] T. Iwashita , I. Yasuda , S. Doi , et al., “Endoscopic Ultrasound‐Guided Antegrade Treatments for Biliary Disorders in Patients With Surgically Altered Anatomy,” Digestive Diseases and Sciences 58 (2013): 2417–2422. 23535877 10.1007/s10620-013-2645-6

[83] K. Minaga , M. Takenaka , K. Yamao , et al., “Clinical Utility of Treatment Method Conversion During Single‐Session Endoscopic Ultrasound‐Guided Biliary Drainage,” World Journal of Gastroenterology 26 (2020): 947–959. 32206005 10.3748/wjg.v26.i9.947 PMC7081009

[84] A. Y. B. Teoh , V. Dhir , M. Kida , et al., “Consensus Guidelines on the Optimal Management in Interventional EUS Procedures: Results From the Asian EUS Group RAND/UCLA Expert Panel,” Gut 67 (2018): 1209–1228. 29463614 10.1136/gutjnl-2017-314341

[85] T. Ogura , N. Nishioka , M. Yamada , et al., “Novel Transluminal Treatment Protocol for Hepaticojejunostomy Stricture Using Covered Self‐Expandable Metal Stent,” Surgical Endoscopy 35 (2021): 209–215. 31932928 10.1007/s00464-020-07381-2

[86] T. Ogura , M. Takenaka , H. Shiomi , et al., “Long‐Term Outcomes of EUS‐Guided Transluminal Stent Deployment for Benign Biliary Disease: Multicenter Clinical Experience (With Videos),” Endoscopic Ultrasound 8 (2019): 398–403. 31552912 10.4103/eus.eus_45_19 PMC6927148

[87] R. Kamiyama , T. Ogura , A. Okuda , et al., “Electrohydraulic Lithotripsy for Difficult Bile Duct Stones Under Endoscopic Retrograde Cholangiopancreatography and Peroral Transluminal Cholangioscopy Guidance,” Gut and Liver 12 (2018): 457–462. 29409310 10.5009/gnl17352 PMC6027838

[88] T. Ogura , W. Takagi , Y. Kurisu , and K. Higuchi , “Technical Tips for Peroral Transluminal Cholangioscopy Using Novel Single‐Operator Cholangioscope (With Videos),” Journal of Hepato‐Biliary‐Pancreatic Sciences 23 (2016): E25–E29. 27531563 10.1002/jhbp.380

[89] A. Hosmer , M. M. Abdelfatah , R. Law , and T. H. Baron , “Endoscopic Ultrasound‐Guided Hepaticogastrostomy and Antegrade Clearance of Biliary Lithiasis in Patients With Surgically‐Altered Anatomy,” Endoscopy International Open 6 (2018): E127–E130. 29399608 10.1055/s-0043-123188 PMC5794438

[90] S. Mukai , T. Itoi , A. Sofuni , et al., “EUS‐Guided Antegrade Intervention for Benign Biliary Diseases in Patients With Surgically Altered Anatomy (With Videos),” Gastrointestinal Endoscopy 89 (2019): 399–407. 30076841 10.1016/j.gie.2018.07.030

[91] Y. Takasaki , S. Ishii , T. Shibuya , et al., “Endoscopic Ultrasound‐Guided Antegrade Procedures for Managing Bile Duct Stones in Patients With Surgically Altered Anatomy: Comparison With Double‐Balloon Enteroscopy‐Assisted Endoscopic Retrograde Cholangiography (With Video),” Digestive Endoscopy 33 (2021): 1179–1187. 33421211 10.1111/den.13927

[92] T. Kanadani , T. Ogura , S. Ueno , et al., “Transluminal Antegrade Drill Dilation Technique for Hepaticojejunostomy Stricture With Cholangioscopic Evaluation (With Video),” Endoscopy International Open 12 (2024): E181–E187. 38348332 10.1055/a-2218-1538 PMC10861321

[93] T. Ogura , J. Matsuno , T. Kanadani , J. Nakamura , and H. Nishikawa , “Antegrade Radial Laser Ablation for a Pancreaticojejunal Stricture Under Peroral Pancreatoscopic Guidance,” Endoscopy 57 (2025): E1192–E1193. 41187779 10.1055/a-2723-1445 PMC12585897

[94] T. Ogura , K. Bessho , N. Hattori , J. Matsuno , and H. Nishikawa , “Technical Tips for Antegrade Endopancreatic Radiofrequency Ablation for Severe Pancreatojejunal Stricture,” Endoscopy 56 (2024): E628–E629. 39059446 10.1055/a-2357-2274 PMC11281845

[95] T. Ogura , Y. Uba , M. Tomita , K. Bessho , and H. Nishikawa , “Endoscopic Ultrasound‐Guided Antegrade Dilation Using a Drill Dilator for Hepaticojejunostomy Stricture With Cholangioscopic Findings,” Endoscopy 55 (2023): E525–E526. 36894151 10.1055/a-2037-5749 PMC9998222

[96] T. Ogura , A. Okuda , M. Imanishi , et al., “Electrohydraulic Lithotripsy for Pancreatic Duct Stones Under Digital Single‐Operator Pancreatoscopy (With Video),” Digestive Diseases and Sciences 64 (2019): 1377–1382. 30456448 10.1007/s10620-018-5374-z

[97] T. W. James and T. H. Baron , “Antegrade Pancreatoscopy via EUS‐Guided Pancreaticogastrostomy Allows Removal of Obstructive Pancreatic Duct Stones,” Endoscopy International Open 6 (2018): E735–E738. 29876510 10.1055/a-0607-2484 PMC5988545

[98] T. Ogura , J. Nakamura , J. Sakamoto , Y. Uba , and H. Nishikawa , “Endoscopic Ultrasound‐Guided Antegrade Dilation Using a Drill Dilator for a Pancreatojejunostomy Anastomotic Stricture, With Pancreatoscopic Findings,” Endoscopy 55 (2023): E617–E618. 37040880 10.1055/a-2055-1306 PMC10089794

[99] M. G. Keane , T. Runge , Y. Ichkhanian , O. Brewer Gutierrez , and M. A. Khashab , “Antegrade Pancreatoscopy With Electrohydraulic Lithotrypsy Through an Endoscopic Ultrasound‐Guided Pancreaticogastrostomy for the Removal of Obstructing Pancreatic Stones,” American Journal of Gastroenterology 117 (2022): 713–714. 35081547 10.14309/ajg.0000000000001654

[100] Z. Hassan and E. Gadour , “Percutaneous Transhepatic Cholangiography vs Endoscopic Ultrasound‐Guided Biliary Drainage: A Systematic Review,” World Journal of Gastroenterology 28 (2022): 3514–3523. 36158274 10.3748/wjg.v28.i27.3514 PMC9346459

[101] Y. Tanisaka , S. Ryozawa , M. Mizuide , et al., “Status of Single‐Balloon Enteroscopy‐Assisted Endoscopic Retrograde Cholangiopancreatography in Patients With Surgically Altered Anatomy: Systematic Review and Meta‐Analysis on Biliary Interventions,” Digestive Endoscopy 33 (2021): 1034–1044. 33073407 10.1111/den.13878

[102] R. Hakuta , T. Sato , Y. Nakai , et al., “Balloon Endoscopy‐Assisted Endoscopic Retrograde Cholangiopancreatography for Hepatolithiasis in Patients With Hepaticojejunostomy,” Surgical Endoscopy 38 (2024): 2423–2432. 38453748 10.1007/s00464-024-10738-6 PMC11078785

[103] K. Nakamura , Y. Ishii , Y. Tatsukawa , et al., “Comparative Study of Therapeutic Outcomes in Patients Who Developed Hepatolithiasis After Hepaticojejunostomy: Balloon‐Assisted Enteroscopic Approach Versus Percutaneous Transhepatic Approach,” Surgical Endoscopy 39 (2025): 1160–1168. 39715955 10.1007/s00464-024-11479-2

